# Behavioural and molecular endophenotypes in psychotic disorders reveal heritable abnormalities in glutamatergic neurotransmission

**DOI:** 10.1038/tp.2015.26

**Published:** 2015-03-31

**Authors:** L Scoriels, R M Salek, E Goodby, D Grainger, A M Dean, J A West, J L Griffin, J Suckling, P J Nathan, B R Lennox, G K Murray, E T Bullmore, P B Jones

**Affiliations:** 1 Department of Psychiatry, University of Cambridge, Cambridge, UK; 2 Instituto de Ciências Biomédicas, Universidade Federal do Rio de Janeiro, Rio de Janeiro, Brazil; 3 Department of Biochemistry, University of Cambridge, Cambridge, UK; 4 European Bioinformatics Institute (EMBL-EBI) European Molecular Biology Laboratory, Wellcome Trust Genome Campus, Hinxton, UK; 5 Index Ventures, London, UK; 6 School of Psychology and Psychiatry, Monash University, Clayton Campus, VIC, Australia; 7 NIHR Biomedical Research Centre, Cambridge, UK; 8 GlaxoSmithKline Clinical Unit Cambridge, Addenbrooke's Centre for Clinical Investigation (ACCI), Cambridge, UK

## Abstract

Psychotic disorders such as schizophrenia are biologically complex and carry huge population morbidity due to their prevalence, persistence and associated disability. Defined by features such as delusions and hallucinations, they involve cognitive dysfunction and neurotransmitter dysregulations that appear mostly to involve the dopaminergic and glutamatergic systems. A number of genetic and environmental factors are associated with these disorders but it has been difficult to identify the biological pathways underlying the principal symptoms. The endophenotype concept of stable, heritable traits that form a mechanistic link between genes and an overt expression of the disorder has potential to reduce the complexity of psychiatric phenotypes. In this study, we used a genetically sensitive design with individuals with a first episode of psychosis, their non-affected first-degree relatives and non-related healthy controls. Metabolomic analysis was combined with neurocognitive assessment to identify multilevel endophenotypic patterns: one concerned reaction times during the performance of cognitive and emotional tests that have previously been associated with the glutamate neurotransmission system, the other involved metabolites involved directly and indirectly in the co-activation of the *N*-methyl-D-aspartate receptor, a major receptor of the glutamate system. These cognitive and metabolic endophenotypes may comprise a single construct, such that genetically mediated dysfunction in the glutamate system may be responsible for delays in response to cognitive and emotional functions in psychotic disorders. This focus on glutamatergic neurotransmission should guide drug discovery and experimental medicine programmes in schizophrenia and related disorders.

## Introduction

Psychotic disorders present a variety of debilitating cognitive and emotional impairments. Problems with memory, cognitive flexibility, ability to plan and emotional perception^1, 2, 3^ often lead to difficulties in social integration and stable employment; this is why psychotic disorders rank number one in global costs for brain disorders in Europe.^4^ Memory, executive function and emotional perception impairments are inherent to the disease, often appear before its onset, and may be present in people genetically related to patients.^5, 6^ These characteristics define what is commonly known as an endophenotype and suggest a genetic predisposition to these symptoms. Multiple genetic studies have been performed to uncover the causes of cognitive symptoms in psychotic disorders, but because of its biological complexity and pronounced heterogeneity, it has been difficult to associate cognitive symptoms to any single gene. The endophenotype concept simplifies this relationship. Cognitive and emotional endophenotypes are the manifestations of abnormalities in neurophysiological systems that are closer to the molecular and genetic mechanisms of the disorder.

Magnetic resonance spectroscopy has shown distortions in specific metabolite levels, such as phosphocreatine, choline or glutamine, in different parts of the brain at the first episode and chronic schizophrenia. These distortions were shown to be directly linked to neurocognitive deficits of the disease.^7, 8, 9^ Metabolic dysregulations do not appear to be solely attributable to antipsychotic treatment and lifestyle but also seem to be inherent biological dysfunctions specific of the disorder^7, 8, 9, 10, 11, 12, 13, 14, 15^ as suggested by studies in relatives of patients with schizophrenia.^16, 17, 18^ Metabolomics is a relatively new technique that analyses the complete collection of metabolites produced by an organism and gives a more dynamic profile compared with proteomics or genomics.^19^ The technique has been applied to white and grey matter postmortem brain tissue in schizophrenia and showed an imbalance in metabolites involved in both glucose and oxidative stress functions in mitochondria in individuals with schizophrenia.^20^ The investigators found the same dysregulations in the cerebrospinal fluid of prodromal and early psychosis patients^21, 22^ and in peripheral blood of patients with schizophrenia,^23^ suggesting that the analysis of non-cerebral biological samples can inform brain states related to schizophrenia. However, this technique has not been applied to relatives of patients with psychotic disorders, or to underlying psychopathological domains. It is not known whether the global metabolic profile in psychosis can be considered as an endophenotype.

In this study, we investigated whether multiple hierarchical endophenotypes link genotype, psychopathology and clinical phenotype, a concept put forward by Craddock and Owen.^24^ First, we assessed whether cognitive and emotional performance and reaction time would show an endophenotypic pattern, which would discriminate individuals with a first episode of psychosis (named hereafter probands) from their first-degree relatives (relatives) and from healthy unrelated controls (controls). First-degree relatives share around 50% of their genetic background with probands and often have increased risk for the disorder. Second, we analysed metabolic profiles in peripheral blood from these three groups to determine whether catecholamine and glutamatergic metabolites also showed endophenotypic patterns. Finally, we investigated the associations between any such endophenotypes.

Our overall strategy indicated that we would find deficits in performance and reaction time in cognitive and emotional tests in probands compared with unrelated controls, with relatives occupying an intermediate position. We also hypothesised that probands, and to a lesser degree their relatives, would present an over-expression of metabolites involved in the dopaminergic neurotransmission system and a hypo-expression of metabolites involved in the glutamatergic neurotransmission system. These represent the two leading neurotransmitter hypotheses of schizophrenia pathophysiology and are supported by recent molecular genetic findings.^25^ Finally, we hypothesised that the endophenotypes in behavioural tests would be associated with an imbalance in metabolic profiles.

We analysed only those cognitive and emotional variables relevant to our hypothesis to reduce the risk of type I error. Furthermore, we used principal component analysis (PCA) to reduce the number and complexity of the metabolic variables.

## Materials and methods

### Study design and sample

The study was a genetically sensitive trio-design of 34 probands with first episode psychosis (FEP), 34 first-degree relatives and 35 unrelated healthy controls. FEP probands were recruited from CAMEO, the early intervention service for psychosis in Cambridgeshire and Peterborough in the United Kingdom (www.cameo.nhs.uk)^26, 27, 28, 29^ and from similar services in London, Suffolk and Nottinghamshire (the study was approved by the Research Ethics Committee in Cambridgeshire, London (06/Q0108/129), Suffolk (07/Q0102/26) and Nottinghamshire (AMH/31/01/08)). Probands were included according to DSM-IV criteria for any psychotic disorder without identifiable medical cause^30^ and had to be within a year of presenting with their first episode. Probands' first-degree relatives included siblings and parents. We included only relatives without psychotic disorder according to DSM-IV. Healthy unrelated control subjects had no history of psychiatric disorder themselves or in their family. Participants were excluded if they had any history of head injury or major learning disability or if they scored positively for standard psychostimulant substances tested in urine samples or had a blood alcohol level above 80 mg l^−1^. Healthy controls were excluded if they had taken psychoactive drugs within 2 weeks before the assessment. Most probands with FEP were taking antipsychotic medication and were asked to continue their treatment for ethical and practical reasons. All the participants provided written informed consent before the study and gave a blood sample and performed a battery of cognitive and emotional tasks during the study. Two probands were excluded for presence of drugs in a urine test and brain abnormality, respectively.

### Serum preparation

Serum samples were collected and stored at −20 °C. None of the samples underwent more than three freeze–thaw cycles before acquisition by nuclear magnetic resonance (NMR) spectroscopy or liquid chromatography coupled with mass spectroscopy. All the experiments were performed under blind and randomised conditions.

### Behavioural analysis

A number of cognitive and emotional domains were assessed. We measured working memory using the letter version of the n-back task, which displays a sequence of different letters and requires participants to press a button whenever they see the same letter as the one seen n-back. Planning abilities were tested with the computerised CANTAB Stockings of Cambridge task, which requires participants to reorganise patterns following a model and a set of rules. Emotion recognition was assessed with the emotion intensity recognition test, which displayed a sequence of faces showing one of the six emotions: happiness, surprise, sadness, fear, disgust and anger. Participants were asked to rate how intense a given facial emotion was. Processing speed of the brain was assessed with the reaction time task, which required participants to press a button as fast as they could when they saw a ball appear in the screen. These tests have been described elsewhere.^27, 31, 32, 33^ One-way or multivariate analysis of variance (ANOVA and MANOVA, respectively) models were used. Normality and homogeneity of data distribution was confirmed using the Shapiro–Wilk and Levine tests. Where appropriate transformations did not result in normal distributions, the non-parametric Kruskal–Wallis test was used. *Post hoc* two-tailed *t*-tests were applied on the measures that led to significant results in the ANOVA or MANOVA. Data were analysed with SPSS software version 15 (Cambridge, UK).

### 1H-NMR spectroscopy acquisition, processing and data reduction

A total 150–200 μl of serum was diluted with 350 μl of 0.9% sodium chloride in water with 10% deuterium for 1H-NMR spectroscopic analysis. 1H-NMR spectra data sets were collected using a Bruker (Cambridge, UK) Avance-III 600 MHz magnet at a nominal proton frequency of 600.13 MHz at an ambient temperature of 310 K, using Bruker TopSpin (version 2.1.3) software for both acquisition and data processing. A standard Carr–Purcell–Meiboom–Gill^34^ pulse sequence was used for one-dimensional data acquisition, with pre-saturation to allow elimination of the solvent signals during the relaxation delay of 1.5 s. An electromagnetic pulse perpendicular (90°) to the main magnetic field was induced to the sample for 90 μs and then a sequence of 63 other perpendicular electromagnetic pulses (180°) with opposite directions was applied with intervals of 1.2 s. Sixty-four scans were collected in total with 32 768 data points in a sweep width of 8389.3 Hz. The free induction decay was therefore 1.95 s. For data processing, the free induction decays were multiplied by a 0.3 Hz exponential weighting function and zero-filled to 65 536 data points before Fourier transformation. Corrections for distortions of the spectra were applied taking lactate (CH_3_=δ 1.33) as a reference. The region around the water peak (δ 5.2−δ 4.4) was excluded, as this signal dominates the spectrum despite solvent suppression due to the abundance of the solvent. The resulting NMR spectra were data reduced (binned) in the region δ 10.0−δ 0.0 in integral segments of equal length (δ 0.01) using the Bruker AMIX software (version 3.8.3). Normalisation to total spectral area was performed by calculating the relative contribution of each bin as a fraction of the total spectral area. Peak alignment and positive and negative control data were checked for the normalised data.

### Liquid chromatography-mass spectrometry

Hundred microlitres of serum was thoroughly dried under nitrogen and derivatised with 400 μl of 3 M HCl in BuOH for 15 min at 65 °C. After further drying, the sample was reconstituted in 9:1 0.1% formic acid in water/acetonitrile and sonicated to ensure solvation of the amino-acid derivatives. Samples were analysed using a Quattro Premier XE MS coupled to an Acquity UPLC system (Waters, Manchester, UK). The strong mobile phase used for analysis was acetonitrile and the weak mobile phase was 0.1% formic acid in water. The analytical UPLC gradient used a HSS T3 column (100 mm × 2.1 mm, 1.7 μm) from Waters with 5% acetonitrile in 0.1% formic acid at 0 min followed by a linear gradient to 40% acetonitrile after 7 min followed by re-equilibration for 3 min. The total run time was 10 min and the flow rate was 0.3 ml min^−1^ with an injection volume of 3 μl. The mass spectrometry parameters were: dwell time 0.02 s, source temperature 150 °C, desolvation temperature 350 °C, capillary voltage 3.5 kV and 700 l h^−1^ of desolvation gas, all other parameters were compound specific.

### Metabolomic analysis

Resulting NMR data were analysed with the SIMCA-P+ software (version 13). Data with no variance were eliminated and the remaining data were winsorised^35^ to ±3 s.d. to moderate the impact of outliers. Pareto scaling^36^ was selected for the models shown here following pragmatic comparison of models built on the unscaled and unit variance scaled data. The NMR spectra were analysed with PCA and partial least square (PLS) models, which are two projection methods that use mathematical algorithms to reduce multivariate data into a few principal components (PCs) that describe the maximum variation within the data. PCA is defined as an orthogonal linear transformation that reduces multidimensional data to a new linear coordinate system such that the greatest variance by any projection of the predicted variables comes to lay on the first coordinate, called the first PC, the second greatest variance on the second coordinate and so on. PCA is independent of observable variables and is mostly used as a tool in exploratory data analysis and for making predictive models. PLS is defined as a linear construct that projects the predicted and observed variables to a new space, specifying the linear relationship between the two observable variables. PLS also produces PCs, which are calculated from the greatest covariance of the predicted and observed variables, and not only the predicted variables, as for PCA. PLS is used as a classification tool for identifying the differences between groups of samples. Here, a PCA was used to reduce the NMR spectrum data into a few PCs that described the maximum variation within the data independently of psychotic hereditary group category. PLS was used with determined group category and enabled the identification of variables related to groups of observation (or classifications). The extent to which PCs from PCA and PLS segregated probands, relatives and controls was tested by comparing the observed distribution of peaks in these three groups with an expected random distribution using *χ*^2^.

A global model was obtained from the PCA and PLS, and cross-validation was applied to test its significance. Cross-validation consisted in repeating the same PCA and PLS analyses as previously, with only 75% of randomly selected samples from each group category. This enabled the construction of a model that was used to predict how the remaining 25% hold-out samples would segregate in the established class models on the basis of their best fit and goodness of prediction to the model. For each subject in the validation set, a predicted Y value (Y') was computed by applying the loadings from the PLS model to the measured X variables for these subjects. To avoid scaling artefacts, and to minimise the impact of arbitrary class cut-offs, predicted class membership was assigned by allocating class 1 membership to the lowest tertile of the Y' values in the hold-out set, class 2 membership to the middle tertile and class 3 membership to the upper tertile. External prediction of the model was performed using *χ*^2^ that compared each predicted group category to the actual group category. A parallel graphical analysis was performed on calculated mean differences between groups. Candidate NMR chemical shift regions were isolated for any difference between these calculated mean differences superior to two standard errors (s.e.). Metabolic candidate compounds were selected using the chemical shift regions from graphical and the PC that showed the greatest variance between all the three groups, and subsequently identified using the Human Metabolome Database (www.hmdb.ca)^37^ and Chenomx NMR Suite (Chenomx version 7.6). Liquid chromatography coupled with mass spectroscopy was performed using the same serum samples and data were analysed using one-way ANOVA statistical technique.

## Results

### Demographics

Demographic analysis showed no difference between groups for age, ethnicity or premorbid IQ. There was a significant difference in the gender ratio between the three groups, due to parents in the relative group being predominantly represented by mothers (Table 1), whereas the proband group was most represented by men. Hence, further statistical analyses included gender as a covariate in all parametric analyses.

### Cognitive and emotional endophenotypes

**Figure 1:**
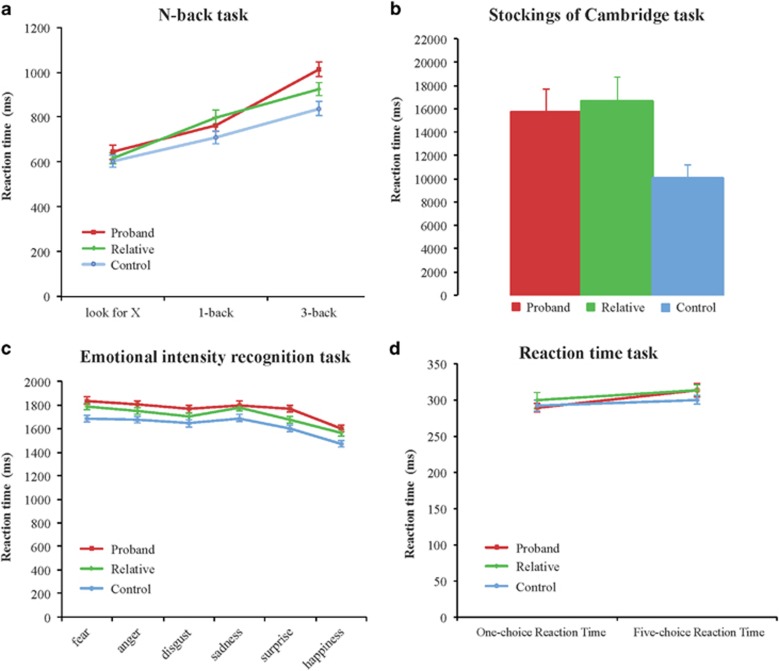
Cognitive, emotional and motoric reaction time responses. (**a**) Reaction time on the n-back task showed that probands (red lines, *n*=32), relatives (green lines, *n*=34) and controls (blue lines, *n*=33) were slower according to task difficulty. The three groups did not present any differences in reaction time for the ‘look for X' and the 1-back tasks (*P*>0.05), but they did for the 3-back task (*P*<0.05), with probands being significantly slower than controls, and relatives having a pattern in between the two. (**b**) Reaction time on the CANTAB Stockings of Cambridge (SOC). Probands (*n*=32) and relatives (*n*=34) were significantly slower compared with controls (*n*=32) (*P*<0.01). (**c**) Latency of each emotion from the Emotion Intensity Recognition Test (EIRT). Latencies in the EIRT task showed that probands were slower at recognising intensities for all emotional faces compared with controls (*P*<0.05). They were also slower than their relatives to recognise surprise. Relatives, in turn, were significantly slower than controls to recognise fear, sadness and happiness facial intensities (*P*<0.05). (**d**) Latency on the CANTAB one- and five-choice reaction time. There were no statistical differences between the three groups (*P*>0.05). Error bars represent s.e.m.

The participants performed a selection of tests of memory, executive and emotional functions. The performance of the n-back task, a test of working memory, showed that the three groups differed statistically significantly for reaction time (*χ*^2^=7.04, *P*=0.03, Kruskall–Wallis test) with probands being the slowest, followed by their relatives; controls were the fastest (Figure 1a). There was a difference for accuracy on the more difficult (3-back) working memory task that reached statistical trend levels (*χ*^2^=5.01, *P*=0.08); probands showed the worst performance, followed by their relatives; controls showed the best performance. The three groups did not differ for the control task (‘look for X') and the easy (1-back) working memory task (Figure 1a). Executive function was assessed with the CANTAB Stockings of Cambridge test that measures cognitive planning abilities. Probands, their relatives and controls differed significantly for their planning reaction time (F_(2,91)_=5.16, *P*=0.008, Figure 1b). *Post hoc t*-tests showed that probands and relatives took significantly more time to plan moves compared with controls (*t*_(30)_=2.22, *P*=0.03 and *t*_(30)_=3.00, *P*=0.004, respectively). The three groups did not differ in the number of problems solved in minimum moves.

The assessment of emotional processing with the emotion intensity recognition task showed differences between the three groups for the reaction time of all emotions (MANOVA, all F_(2,96)_>3.50 and *P*<0.05, Figure 1c). *Post hoc t*-tests showed that probands were performing significantly slower compared with controls for all reaction times of emotion intensity recognition task (all *t*_(30)_>2.5, *P*<0.05). Relatives showed significant increase in reaction time compared with controls for fear (*t*_(30)_=2.68, *P*=0.01), sadness (*t*_(30)_=2.15, *P*=0.04) and happiness (*t*_(30)_=2.43, *P*=0.02). There was a significant difference in reaction time for surprise intensity recognition between probands and relatives (*t*_(30)_=2.33, *P*=0.02, Figure 1c). The three groups did not differ significantly for accuracy in the identification of emotional intensity.

Tests of purely motoric functions, the one- and five-choice reaction time tests, showed no statistical differences between the three groups (Figure 1d).

### Metabolic endophenotypes

We used proton NMR spectroscopy (1H-NMR) to investigate whether potential endophenotypes were related to metabolic dysfunction. The initial NMR data set was composed of 1026 bin separated regions for each participant, reduced to 365 after winsorisation and elimination of data with no variance (small signal-to-noise ratio). The data set was analysed using multivariate approaches with algorithms and projection-based methods aimed at reducing the dimensionality of the data. PCA, an unsupervised method (independent of the group classification) and partial least square discrimination analysis (PLS-DA), a supervised approach (dependent on the group classification) were used to analyse the data set. PCA allows analysing the NMR-derived metabolic profiles for bin regions representing chemical peaks of organic compounds. Both PCA and PLS methods facilitate the identification of candidate compounds, levels of which may vary between the three groups (probands, relatives and controls) according to psychosis hereditary association.

A PCA model was built using 10 components, which explained ~70% of the variance (*R*^2^(X)=0.69) and had a predictability of 53% (*Q*^2^(X)=0.53). Analysis of the PCs by group classification showed that the seventh component (PCA-PC7) was responsible for the best segregation of bin regions between FEP probands, their first-degree relatives and healthy controls (*χ*^2^=16.92, *P*=0.0002). The third component (PCA-PC3) also segregated bin regions differentially, depending on the group classification, although more moderately (*χ*^2^=6.83, *P*=0.03). No other PC showed statistically significant differences between the groups (*P*>0.05). The segregation was such that metabolic profiles from probands in PCA-PC7 were in one extremity of the plot and controls on the other extremity, with relatives half way through probands and controls metabolic profiles.

**Figure 2:**
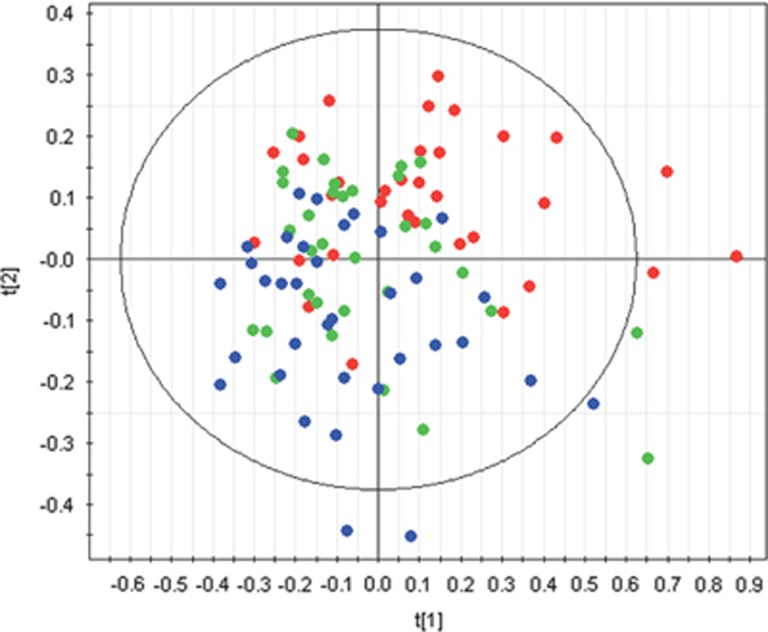
Groups distribution of nuclear magnetic resonance (NMR) chemical shift regions in components from the global partial least square (PLS) model. Red circles represent NMR chemical shift distribution in probands (*n*=34), green circles NMR chemical shift distribution in relatives (*n*=33) and blue circles NMR chemical shift distribution in controls (*n*=35). Component 2 shows a significant split between probands, relatives and controls (*χ*2=19.05, *P*=0.00007). Component 1 also shows an effect of group in the segregation of peaks to a more moderate level (*χ*2=9.62, *P*=0.01).

A global PLS model (PLSg) was first built with all the data. Data auto-fitted a PLS model with two components, with a predicted best fit of *R*^2^(X)=0.38. Analysis of the bin regions by group classification showed that the second PC (PLSg-PC2) was responsible for the best statistically significant segregation of bin regions distribution between probands, relatives and controls (*χ*^2^=19.06, *P*=0.00007) and the pattern was the same as the one seen in the PCA model. The first component (PLSg-PC1) also significantly segregated bin regions between groups, but the effect was smaller than for PLSg-PC2 (*χ*^2^=9.62, *P*=0.01, Figure 2).

A new PLS model was built for external validation (PLSev) with 75% of randomly selected participant samples from each group. PLSev was optimised for a model containing 9 PCs, which showed similar patterns of segregation between metabolic bin regions from probands, their first-degree relatives and controls, with relatives in an intermediate position (PLSev-PC2 (*χ*^2^=23.44, *P*=0.000008), PLSev-PC3 (*χ*^2^=18.96, *P*=0.00008), PLSev-PC1 (*χ*^2^=8.89, *P*=0.01)). External validation with the remaining 25% of the data showed the same pattern but did not reach statistical significance (*P*=0.24).

Since the PCA model suggests an endophenotypic separation by group classification in an unsupervised manner (that is, that does not consider the groups classification when calculating the PCs), this model was used to select endophenotypic metabolic candidates. All bin regions found to be in excess of expected frequency from a Gaussian distribution in PC7 were identified. Differences in distribution calculated between probands and their relatives, probands and controls and relatives and controls were analysed graphically in an eigen-spectrum (Supplementary Table 1).

### Identification of metabolites converges on the glutamatergic biological pathway

The Human Metabolome Database and Chenomx NMR Suite (Chenomx version 7.6) were used to search and identify the chemical shifts found both from PCA-PC7 and the graphical analyses. Five metabolic candidates were annotated on the basis of the NMR chemical shift regions that were over-expressed in probands: α-aminocyclo-propanecarboxylate (ACPC), lactate, acetate, 1-hydroxyisobutyrate and octane. ACPC is a partial agonist/antagonist of the glycine cotransmitter site of the *N*-methyl-D-aspartate (NMDA) receptor.^38^ L-lactic and acetic acid are components of the glycolytic metabolic pathway.^39^ However, no metabolic functions have yet been assigned to 1-hydroxyisobutyrate and octane. The bin regions that were under-expressed in probands were much more abundant and led to 10 metabolic candidates: D-serine, glycine, betaine, guanidoacetate, dimethylsulfide, phosphocreatine, pyruvate, 1,9-dimethyluric acid, trimethylamine oxide and diethanolamine (Table 2).

Glycine and D-serine are both co-activators of the glycine cotransmitter site of the NMDA receptor.^40^ Betaine, guanidoacetate, dimethylsulfide, phosphocreatine and pyruvate are metabolites involved in the anabolism and catabolism of glycine and serine via the methionine to cysteine metabolism in astrocytes (cells that provide nutrients, neurotransmitters and co-activators to synapses).^41^ Trimethylamine oxide is also likely to be related to this metabolism, as it has been shown that betaine can produce it in two chemical reactions steps in marine animals;^42^ however, data in humans are not available. 1,9-Dimethyluric acid is involved in the metabolism of urine, purines and caffeine,^43^ but no connections with metabolic dysfunctions in psychosis have yet been reported. Diethanolamine is an exobiotic compound that can be metabolised by biosynthetic routes common to ethanolamine, which again is involved in glycine and serine metabolism, albeit more remotely.^44^

Liquid chromatography coupled with mass spectroscopy was performed to verify the identity of the main candidate metabolites from the NMR, that is, metabolites involved in the glutamatergic system. One-way ANOVA did not show statistically significant differences between groups for levels of glycine (F=0.76, *P*=0.47), serine (F=1.26, *P*=0.29), ACPC (F=0.10, *P*=0.91) or betaine (F=1.30, *P*=0.28).

### Two endophenotypes but a single construct?

We performed two-tailed Pearson correlations between the scores obtained in cognitive and emotional reaction time tasks and the intensity of the NMR chemical shift regions from the candidate metabolites that segregated the three groups to the greatest degree. Only three negative correlations were found between the bin region corresponding to dimethylsulfide (2.095) and the reaction time to identify sad, surprised and happy faces intensities (*r*=−0.21, *P*=0.04 for the three emotion intensities).

## Discussion

This study shows linearity in reaction time responses related to cognition and emotion in probands, relatives and controls that are consistent with endophenotypic characteristics. Probands, and to a lesser extent, relatives respond to cognitive and emotional tests more slowly than healthy, unrelated controls. Slower reaction time may have a deleterious impact on subjects' day-to-day function despite having little effect on performance in the controlled context of the experimental paradigm. Indeed, it may well be that patients develop performance impairments as a consequence of increased latencies in several domains of their lives, *in situations* dependent upon optimal cognitive and emotional abilities.

Linearity is also observed in the distribution of the metabolic profile of the three group categories. Probands show a distribution of chemical peaks in one extreme of the PLS score plot, whereas controls show a distribution that spreads in the opposite direction. The chemical peaks from probands' first-degree relatives overlap with the two other groups' distributions. These variations in chemical shift distribution between the three groups appeared to be mostly due to changes in the glutamatergic neurotransmission system in general, and the anabolism and catabolism of serine and glycine, in particular.

**Figure 3:**
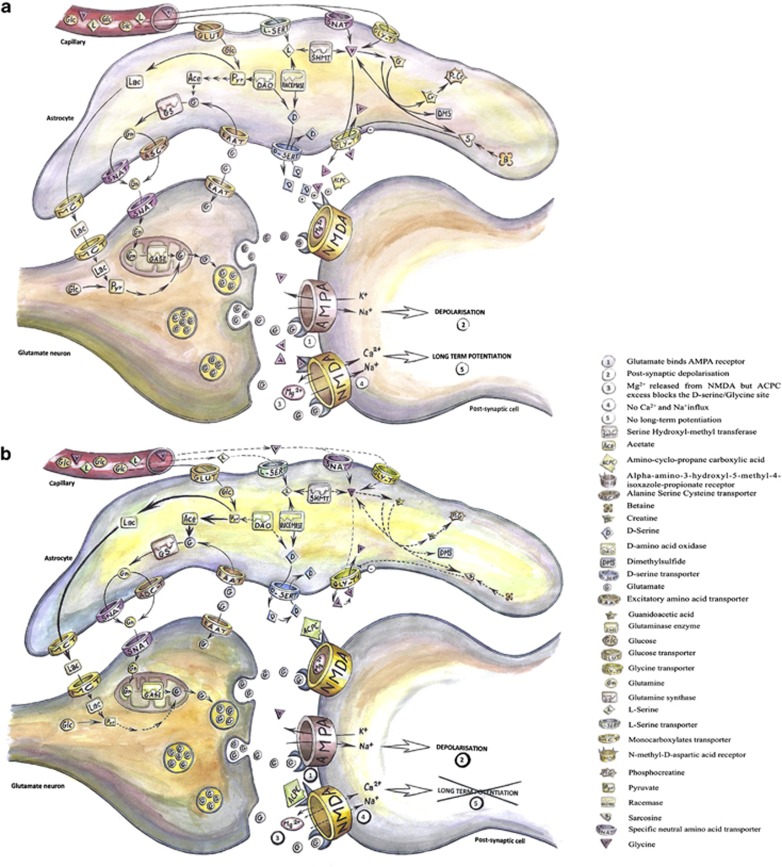
Role of the metabolites found in the metabolomics technique. (**a**) Normal glutamate neuron synapse. Glutamate from the pre-synaptic cell binds the post-synaptic AMPA and NMDA receptors. Na^+^ and K^+^ ions enter the post-synaptic cell, via AMPA receptors, creating a depolarisation. Then, NMDA receptors that have bound glutamate and the co-activator glycine or D-serine, which comes from the surrounding astrocytes, free their channel from Mg^2+^, allowing Na^+^ and Ca^2+^ to enter the post-synaptic cell, which creates a long-term potentiation. After the signal is transmitted, glutamate is re-uptaken by excitatory amino acid transporter (EAAT) located in the pre-synaptic cell and astrocytes. Glutamate that enters the astrocytes is transformed in glutamine by the glutamine synthase and is transported to the pre-synaptic cell via alanine serine cysteine transporter (ASCT) or specific neutral amino acid transporter (SNAT) and re-synthesised in glutamate because of the glutaminase enzyme. Glycine can come from the vascular system via glycine transporters (GLY-T1) or SNAT, or from its metabolism, from transformation of L-serine with the serine hydroxyl-methyl transferase (SHMT) or from sarcosine that involves methylation and demethylation processes. Glycine is released to the synapse interspace via GLY-T1. The D-serine is synthesised from L-serine with the racemase enzyme and released to the synapse interspace by the D-serine transporter (D-SERT). The L-serine can be brought by the vascular system via the L-serine transporter (L-SERT) or be synthesised from glycine with the SHMT. The D-serine can also be synthesised from pyruvate via the D-amino acid oxidase (DAO). The NMDA glycine/D-serine co-activation sites can also bind to a less extent α-amino cyclopropane carboxylic acid (ACPC), which is a partial agonist of this site. (**b**) Metabolic dysfunction that may occur in the glutamate synapse in first episode psychosis. Metabolism involved in the synthesis or transport of glycine and D-serine appears to be down-regulated, inducing a reduction or a lack of glycine and D-serine in the synapse interspace. As a consequence, ACPC can bind the NMDA co-activator site, but because of its reduced affinity, the NMDA receptor has a reduced activity or fails to induce a long-term potentiation in the post-synaptic cell.

In the normal glutamatergic synapse system, vessels supply astrocytes surrounding the synapse with glycine and L-serine. These are then metabolised and glycine and D-serine are released in the inter-synaptic space.^40^ When the pre-synaptic neuron releases glutamate, it binds to NMDA and AMPA receptors. AMPA receptors are activated and create a depolarisation in the post-synaptic cell. This depolarisation, combined with glutamate and glycine/D-serine binding to NMDA receptors^45^ induces a long-term potentiation in the post-synaptic cell, which consolidates neuronal signalling.^40^ The signalling ends when neurons and astrocytes re-uptake neurotransmitters and co-activators. Astrocytes are efficient in the re-uptake of these compounds, which enter the cell metabolism and are recycled for future signalling (Figure 3a). The metabolic candidate compounds found in this study suggest that the metabolism involved in the synthesis or transport of glycine and D-serine may be down-regulated, inducing a reduction or a lack of glycine and D-serine in the glutamate neuron synapse interspace. As a consequence, ACPC, which appears to be overly abundant, binds the NMDA co-activator site and because of its reduced affinity for the site,^46^ ACPC may actually act as an antagonist of the NMDA receptor,^47^ causing a reduced activity or a failure to induce a long-term potentiation in the post-synaptic cell (Figure 3b).

The under-expression of glycine and D-serine in patients with a first episode of psychosis is consistent with a hypofunction of the NMDA receptor, which is one of the theories explored to explain the neurophysiopathology of schizophrenia,^48^ and supports studies that show the efficiency of glycine treatment in negative and other psychiatric symptoms in schizophrenia.^49^ Importantly, this study provides strong, though not yet definitive, evidence that the metabolic dysfunctions observed in probands are not only limited to the NMDA receptor co-activators themselves, but to metabolic pathways involving their anabolism and catabolism via the methionine and cysteine system. These dysfunctions follow an endophenotypic pattern, as relatives show imbalances that are intermediate between probands and controls.

It is known that the NMDA receptor is involved in several cognitive functions, and in memory in particular.^50^ Thus, the question that followed our investigation is whether the endophenotypic patterns of imbalances observed in metabolites were at all associated with the endophenotypic patterns found in cognitive and emotional reaction times in FEP probands. The correlations between the two endophenotypes were weak, probably because of the mechanistic distance between them. However, the literature suggests that hypofunction of the NMDA receptor induces a slowing of reaction time in tasks related to cognition but not motor functions.^51, 52^ This is exactly what our data show, although replication is needed with a larger sample size to specify the effect more precisely. Reaction times related to memory, cognitive flexibility and emotion recognition were increased in probands and to a lesser extent, in relatives compared with controls; however, reaction times that did not involve cognitive or emotional functions, did not show this pattern, suggesting that the endophenotypic imbalance found in the glutamatergic system may be causing the impairments in reaction times related to cognition and emotion functions, albeit remotely. We believe this is the first time that endophenotypes at different levels, psychological and molecular, appear to form a single construct.

There are caveats to this study. First, the sample size for this study was at the lower limit for a differential detection of metabolites between the three groups. Nevertheless, if the peaks identified in the metabolomics analysis had been statistical quirks due to the small sample size, identified only as a result of model over-fitting, then there is little chance they would have, again by chance, been associated with a common metabolic pathway, that is, the NMDA receptor co-activators metabolism. The difficulty in identifying these individual metabolites with liquid chromatography coupled with mass spectroscopy demonstrates discrepancies between the two techniques that have also been shown in other studies^53^ and points out the technical and statistical challenges that are yet to be overcome in terms of finding biological markers in psychiatric disorders. For this reason, increasingly formalised ‘pathway analysis' has gained in popularity as an approach to multivariate modelling of metabolomic data sets and this kind of analysis provides robust validation of the multivariate modelling. It seems very likely that the metabolic imbalance leading to decreased levels of NMDA receptor co-activators is an important pathway predisposing to FEP, and future studies should focus on testing this proposition.

Second, we need to consider the possibility that effects of antipsychotic drug treatment may have affected our results. We think the endophenotypic pattern is unlikely to have been caused in this way because drug treatment in probands with psychosis would not explain the intermediate position of unaffected relatives between probands and healthy unrelated controls. Nevertheless, the impact of treatment on the metabolic signature identified cannot be completely excluded. Indeed, animal studies have shown that typical (haloperidol) and atypical (clozapine) antipsychotics modulate NMDA receptor function.^54^ However, the protective effects of these agents against NMDA receptor antagonists (MK-801), which produce psychosis-like symptoms, suggest that the antipsychotic medication induce NMDA receptors and do not down-regulate them.^55^ Similarly, clozapine, like D-serine, enhances social memory in rats,^56^ suggesting that clozapine is likely to increase levels of D-serine rather than reduce it. Thus, it is highly improbable that the metabolic dysfunction presented in this study is related to medication intake, and the proposition that the only effective treatments for psychosis increase NMDA receptors only underlines further the plausibility of a molecular signature of FEP involving loss of NMDA receptor co-activators. Furthermore, NMR signals from medication-related metabolites were analysed and compared against the list of metabolic candidates that were revealed from the metabolomic analysis. Medication did not appear to have ‘contaminated' the results, as the candidate metabolites presented here were linked to different compounds than those present in the products of antipsychotic drugs.

Despite these caveats, it was striking that the cognitive and emotional impairments in psychosis and the under-expression of NMDA receptor co-activators all followed an endophenotypic pattern. This further supports the model of hypofunction in this receptor as a key step in the pathogenesis of psychosis.^57^ The link between NMDA receptors and both immunologic^58^ and accelerated ageing processes^59^ implicated in psychosis. The immune system is actually highly involved in the pathophysiology of schizophrenia and also deserves further attention.^60, 61^

The endophenotype concept gives the opportunity to overcome current technological issues and provides answers concerning the biological mechanisms involved in the etiophysiopathology of psychotic disorders. Thus, in light of these results, we suggest that new neuropsychological and neuropharmacological treatments targeting cognitive and emotional impairments in psychosis should be developed in the context of abnormalities in the glutamatergic neuronal system.

## Figures and Tables

**Table 1:** Participants demographic information

	*Probands* n*=34*	*Relatives* n*=34*	*Controls* n*=35*	P*-value*
Age (mean years±standard error)	23±1	31±2	27±2	0.27
Gender (men, women)	**24, 10**	**14, 20**	**21, 12**	**0.04**
Ethnicity (white European, others)	30, 4	30, 4	33, 2	0.91
Estimated verbal IQ (National Adult Reading Test) (mean score±standard error)	111±1	110±1	108±1	0.24
Body mass index (mean score±standard error)	25±1	24±1	25±1	0.20

Abbreviation: IQ, intelligence quotient.

Bold values are statistically significant.

**Table 2:** Candidate metabolites over- and under-expressed in probands

*Candidate metabolic compounds with under-expressed peaks*	*Candidate metabolic compounds with over-expressed peaks*	*Metabolic function*
**Serine** **Glycine**	**α−aminocyclo-propanecarboxylate**	Metabolites involved in the activation of the NMDA receptor
*Guanidoacetate* *Dimethylsulfide* *Phosphocreatine* *Betaine* Diethanolamine Trimethylamine oxide		Metabolites involved in the anabolism and catabolism of glycine and serine via the methionine to cysteine metabolism
*Pyruvate*	*Lactate* *Acetate*	Metabolites involved in the oxygenation system
1,9 Dimethylurate		Metabolite involved in the urine, purine and caffeine metabolism
	Hydroxyisobutyrate Octane	Compounds related to petrol

Abbreviation: NMDA, *N*-methyl-D-aspartate.

Metabolic candidate compounds directly involved in the NMDA glycine cotransmitter site are in bold.

Metabolic candidate compounds indirectly involved in the NMDA glycine cotransmitter site are in italics.

## Notes

AMD received a studentship from GlaxoSmithKlein (GSK). PJN was employed by and held shares in GSK. ETB is employed and holds shares in GSK. PBJ has been a member of a Scientific Advisory Board for Roche in 2013. The remaining authors declare no conflict of interest.
