# Supplementary material for: Behavioural and molecular endophenotypes in psychotic disorders reveal heritable abnormalities in glutamatergic neurotransmission
Source: Transl Psychiatry. 2015 Mar 31;5(3):e540–. doi: 10.1038/tp.2015.26 (PMC4429170; doi:10.1038/tp.2015.26)
Supplement: Supplementary Table 1 [file tp201526x1.doc]

**Supplementary Table 1.** **Probands peaks over- or under-expression from PCA and graphical analysis**

| NMR chemical shift regions from PC7 | | NMR chemical shift regions from graphical analysis | |  |
| --- | --- | --- | --- | --- |
| Over-expressed | Under-expressed | Over-expressed | Under-expressed |  |
| ***0.905*** | **1.465** | *0.885^s^* | 0.975 |  |
| ***0.915*** | ***2.095*** | *0.895^s^* | 1.045 |  |
| *0.925* | ***2.105*** | ***0.905^s^*** | **1.465^s^** |  |
| *0.935* | ***2.115*** | ***0.915^s^*** | 2.045 |  |
| *0.945* | *2.425* | **1.205** | ***2.095*** |  |
| 0.985 | *2.435* | *1.215* | ***2.105*** |  |
| **1.205** | *2.445* | *1.225* | 3.045 |  |
| **1.275** | **3.205** | *1.235* | **3.205** |  |
| 1.925 | *3.545* | 1.265 | *3.225* |  |
|  | ***3.555*** | *1.285^s^* | *3.235* |  |
|  | 3.595 | *1.295^s^* | *3.245* |  |
|  | *3.745* | *1.305^s^* | 3.355 |  |
|  | *3.755* | *1.315^s^* | 3.485 |  |
|  | *3.765* | *1.325* | **3.555** |  |
|  | *5.375* | *1.335* | 3.585 |  |
|  | *5.385* | 1.345^s^ | *3.705* |  |
|  |  | 1.485 | *3.715* |  |
|  |  | 2.375^s^ | 3.725 |  |
|  |  | 4.115 | 3.785 |  |
|  |  |  | 3.845 |  |
|  |  |  | *3.895* |  |
|  |  |  | *3.905* |  |
|  |  |  | *3.915* |  |
|  |  |  | *3.925* |  |
|  |  |  | *3.935* |  |
|  |  |  | 3.955 |  |
|  |  |  | 5.245 |  |
| **bold** = common bin regions found in PCA and graphical analyses | | | | |
| ^s^ = shift to the left for proband or proband and relative | | | | |
| *italics* = contiguous bin regions | | | | |
